# Network Model With Reduced Metabolic Rate Predicts Spatial Synchrony of Neuronal Activity

**DOI:** 10.3389/fncom.2021.738362

**Published:** 2021-10-07

**Authors:** Pangyu Joo, Heonsoo Lee, Shiyong Wang, Seunghwan Kim, Anthony G. Hudetz

**Affiliations:** ^1^Center for Consciousness Science, Department of Anesthesiology, University of Michigan, Ann Arbor, MI, United States; ^2^Department of Physics, Pohang University of Science and Technology, Pohang, South Korea

**Keywords:** neuronal network, computational model, brain metabolism, synchronization, anesthesia

## Abstract

In a cerebral hypometabolic state, cortical neurons exhibit slow synchronous oscillatory activity with sparse firing. How such a synchronization spatially organizes as the cerebral metabolic rate decreases have not been systemically investigated. We developed a network model of leaky integrate-and-fire neurons with an additional dependency on ATP dynamics. Neurons were scattered in a 2D space, and their population activity patterns at varying ATP levels were simulated. The model predicted a decrease in firing activity as the ATP production rate was lowered. Under hypometabolic conditions, an oscillatory firing pattern, that is, an ON-OFF cycle arose through a failure of sustainable firing due to reduced excitatory positive feedback and rebound firing after the slow recovery of ATP concentration. The firing rate oscillation of distant neurons developed at first asynchronously that changed into burst suppression and global synchronization as ATP production further decreased. These changes resembled the experimental data obtained from anesthetized rats, as an example of a metabolically suppressed brain. Together, this study substantiates a novel biophysical mechanism of neuronal network synchronization under limited energy supply conditions.

## Introduction

Neuronal activity in the brain is tightly coupled to the level of cerebral energy metabolism. An increased brain metabolic rate leads to a rise in spiking activity (Smith et al., 2002; Mäkiranta et al., 2005). When energy metabolism is diminished, the spontaneous spiking activity in the cortex and several subcortical areas, such as the thalamus and striatum, slows down, exhibiting rhythmic bursts. In electroencephalogram (EEG) recordings, firing bursts are reflected in the appearance of slow (0.1–1 Hz) oscillation or, in more deeply suppressed states, by burst suppression, a phenomenon of transient electrocortical activity alternating with electrical silence. These phenomena are commonly observed in general anesthesia, comas, and hypothermia (Brown et al., 2010; Westover et al., 2015), all of which are associated with reduced brain metabolism.

Several prior studies have attempted to computationally model the effect of varying cerebral energy metabolism on neuronal dynamics consistent with experimental observations. Cunningham et al. replicated experimental findings by an excitatory neuronal network with ATP-gated potassium channels to show the emergence of a slow oscillation pattern when the ATP production rate was downregulated (Cunningham et al., 2006). Likewise, burst suppression was effectively modeled with an interaction between neuronal dynamics and brain metabolism (Ching et al., 2012).

The degree of synchronization of the firing of neurons is an important determinant of information processing in the neuronal network (Plankar et al., 2013). Synchrony may indicate the functional communication of neurons, but it may also mean diminished coding diversity. How the firing synchrony among proximal neurons vs. distant neurons is affected under varying metabolic rates has not been determined. Although both slow oscillation and burst suppression arguably arise from the locally synchronous activity of a neuronal ensemble, these states can also be either globally synchronized or desynchronized. For example, slow oscillations under propofol anesthesia are known to be locally synchronized and globally desynchronized (Lewis et al., 2012; Flores et al., 2017).

Computational modeling of neuronal network dynamics can help answer these questions. In order to examine the effect of the brain metabolic rate on spatial synchronization, we developed a novel neuronal network model that incorporated a varying rate of ATP production. This model allowed us to examine the effect of cerebral hypometabolism on the synchronization pattern of neuronal firing at various spatial scales and helped in the discovery of the potential mechanisms of the dynamic transition among different firing states. As we show here, lowering the ATP production rate in the model leads to a reduced firing rate and increased spike synchronization that develops first locally and with a greater reduction of ATP production, also globally. The results reproduce experimentally observed changes in the neuronal network activity, including spatial synchrony and a spike rate of population activity, as observed under anesthesia.

## Materials and Methods

### Model

#### Leaky Integrate-and-Fire Neuron With ATP Dynamics

The ATP-gated potassium channel has been suggested as a key component in networks of Hodgkin–Huxley-type neurons that exhibit metabolism-dependent slow oscillation and burst suppression (Cunningham et al., 2006; Ching et al., 2012). To efficiently simulate metabolic-dependent slow activity in a large network, we constructed a simplified neuronal model by adding an ATP-dependent current term that behaves like the ATP-gated potassium channel in leaky integrate-and-fire neurons. The membrane voltage-current equation is

$$\begin{array}{l} c_{i}\frac{dv_{i}}{dt}=I_{app,i}+I_{leak,i}+I_{ATP,i}+\underset{j}{\sum }C_{ij}I_{syn,j\to i},\\ I_{app,i}=N\left(I_{app0},\sigma_{I}^{2}\right),\\ I_{leak,i}=-v_{i}/\tau_{leak}, \end{array}$$

where *I*_*app*_ is an externally driven Gaussian noise current with a mean *I*_*app*0_ = 0.03 and SD σ_*I*_ = 0.006, *I*_*leak*_ is leakage current with a time constant τ_*leak*_ = 38.75 ms (Lansky et al., 2006), *I*_*ATP*_ is the ATP-dependent current term, *I*_*syn*_ is the synaptic current, and *C*_*ij*_ is a constant of synaptic strength from the *j*th neuron to the *i*th neuron. *v* is defined to range from 0 (reset) to 1 (threshold), and the capacitance *c*_*i*_ is defined as 1.

The incoming synaptic current *I*_*syn*_ induces a positive perturbation on the membrane voltage *v* and is defined by the following equation:

$$\begin{array}{l} I_{syn,j\to i}\left(t\right)=\underset{spike}{\sum }\frac{\left(t-t_{spike,j}\right)}{\lambda }e^{-\frac{t-t_{spike,j}}{\lambda }}. \end{array}$$

The functional form of the postsynaptic current follows $\frac{t}{\lambda }e^{-t/\lambda }$, where the decay time constant of excitatory postsynaptic potential (EPSP) is λ = 2 ms. For example, if the synaptic strength for a linked synapse is *C*_*ij*_ = 1, the cumulated effect of *C*_*ij*_*I*_*syn, j*→*i*_ on *v*_*i*_ from a single spike is 2.

The dynamical equations for *I*_*ATP*_ are

$$\begin{array}{l} \;I_{ATP,i}=-\alpha \frac{v_{i}}{{\left[ATP\right]}_{i}/{\left[ATP\right]}_{\max}},\\ \frac{d{\left[ATP\right]}_{i}}{dt}=\frac{{\left[ATP\right]}_{\max}-{\left[ATP\right]}_{i}}{\tau_{ATP}}-\varepsilon \delta \left(t-t_{fire,i}\right), \end{array}$$

where α is the conductance of the ATP-gated potassium channel, ε is the ATP consumption per each spike, and τ_*ATP*_ is the time constant for ATP production from mitochondrial energy production. The parameter values for α = 0.002, ε = 0.005, and [*ATP*]_max_ = 1.

#### Mean-Field Feedback Model

We first analyze the mean-field feedback model of a single neuron to identify the most common dynamical patterns that can emerge from the neuron model we use. That is, we examined the single neuron dynamics that eliminate the effect of a network structure. In the voltage-current equation, the term corresponding to the synaptic input from the network is simplified in the mean-field feedback model. The membrane voltage-current equation was computed by the following equations.

$$\begin{array}{l} \;c\frac{dv}{dt}=I_{app}+I_{leak}+I_{ATP}+C_{feedback}iFR_{MF}\left(t\right),\\ iFR_{MF}\left(t\right)=\left[\int_{t-L}^{t}w_{0}\left(t^{\prime }-t+L\right)\underset{spike}{\sum }\delta \left(t^{\prime }-t_{spike}\right)dt^{\prime }\right]\\ \;/\left[\int_{0}^{L}w_{0}\left(t^{\prime \prime }\right)dt^{\prime \prime }\right]. \end{array}$$

In the mean-field feedback model, *C*_*ij*_, which corresponds to the synaptic strength of the neural network, is replaced by a constant *C*_*feedback*_ = 0.4, consistent with the network model; *I*_*syn*_ is replaced by the own firing rate *iFR*_*MF*_ (instantaneous firing rate of the mean-field feedback model) of the neuron. This feedback firing rate is calculated with a Hann window (*w*_0_) of 200-ms length (*L*) at every time step. In addition, we excluded the deviation of *I*_*app*_ here to eliminate possible stochastic variability.

#### Network Architectures

**Figure 1:**
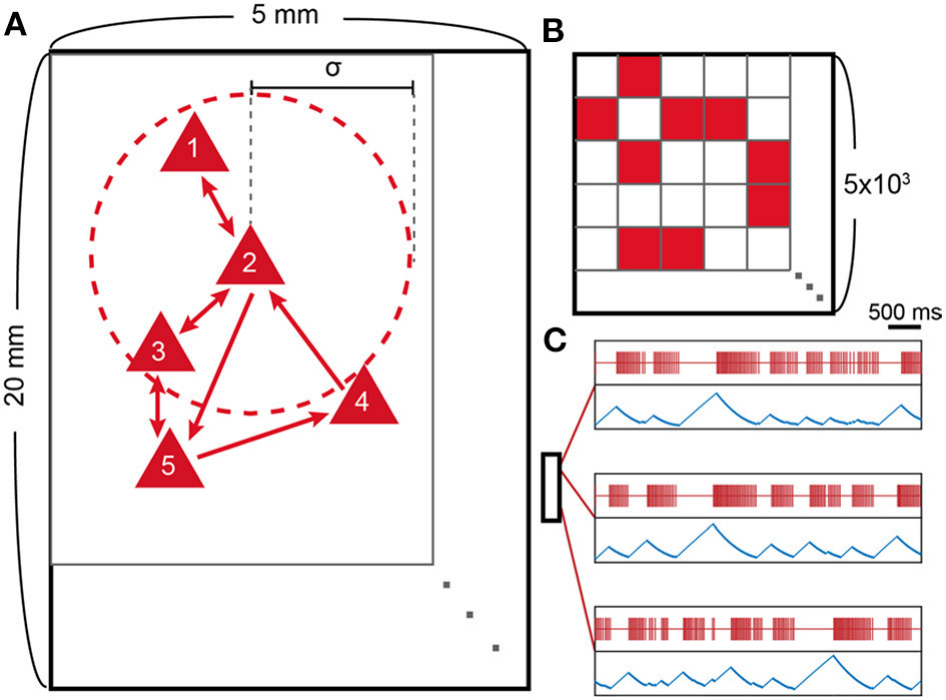
Model schematic and spike patterns. **(A)** Schematic representation of the neuron network model. Red triangles represent excitatory neurons, and outer red circles (dotted line) around neuron 2 represent the interaction range of neuron 2, which is characterized by the SD (σ = 250 μ*m*) of the Gaussian probability distribution. **(B)** Connectivity matrix (*C*_*ij*_; 5 × 10^3^ by 5 × 10^3^) of the schematic. The synaptic connection is made from *j* to *i*, where *i* is row number and *j* is column number. **(C)** Location-dependent spike patterns of the model. Red lines represent single neuron spikes, and blue lines show the corresponding ATP level of the same neuron. The small box represents the 20 × 5 mm rectangular area with three selected neurons according to their locations. The two uppermost neurons are selected from nearby locations and, therefore, show similar spike patterns.

We constructed a 2D network of 5,000 leaky integrate-and-fire neurons (Figure 1). The neurons are randomly scattered on a 2D rectangular area (5 × 20 mm) and make contacts to nearby neurons with a probability that depends on the spatial distance between two neurons. The probability distribution follows a Gaussian distribution, which is centered at the origin and has an SD of σ = 250 μm (Compte et al., 2003), and the resulting node degree is 10 ± 2.85 (SD). *C*_*ij*_ is normalized so that the sum of the incoming synaptic strength could have an equal value of 0.4 for each neuron (i.e., $\underset{j}{\sum }C_{ij}=\;0.4$). A rectangular arrangement, instead of a square one, is used to efficiently simulate the correlations among distant neurons.

#### Simulated LFP

We computed the simulation of local field potential (sLFP) in order to characterize the collective behavior of the system and compare it to the experimental data. The sLFP is calculated by summing up the excitatory postsynaptic currents (EPSC) weighted by a shape function *f*(*l*),

$$\begin{array}{l} \text{sLFP}=-\sum EPSC\times f\left(l\right). \end{array}$$

Here, *f*(*l*) is a weighting function that only depends on the distance (*l*) between the measuring point and a neuron, which represents a single neuron contribution to LFP. A detailed study has been conducted on the form of the shape function (Lindén et al., 2011), where we use the following distance dependency; *f*(*l*) is flat when *l* < 100 *μm* and follows *l*^−2^ scaling when *l* > 100 *μm*. We added a negative sign so that the sLFP has a large negative value when the firing rate is high, as observed in a typical LFP.

#### Numerical Method

The numerical simulation was performed in MATLAB with a 0.5 ms time step using a second-order Runge–Kutta method. The simulations were run for a time period of 120 s, and the data for the first 20 s were removed from the analysis to avoid undesired transient effects.

### Anesthesia Experiment

#### Experimental Procedures

The study was approved by the Institutional Animal Care and Use Committee in accordance with the Guide for the Care and Use of Laboratory Animals of the Governing Board of the National Research Council (National Academy Press, Washington, DC, 2011). The experimental data used in this study were previously analyzed and published in a different context (Lee et al., 2020, 2021). A multi-electrode array consisting of 64-contact silicon probes (shank length 2 mm, width 28–60 μm, probe thickness 15 μm, shank spacing 200 μm, row separation 100 μm, contact size 413 μm^2^; custom design 8 × 8_edge_2 mm 100_200_413; Neuronexus Technologies, Ann Arbor, MI, United States) was chronically implanted in the primary visual cortex of each animal (eight adult male Long-Evans rats). The tips of the probes were placed 1.6 mm below the pial surface. For the recording of an electromyogram, a pair of insulated wires (Plastics One, Inc., Roanoke, VA, United States) exposed at their tip was placed bilaterally into the nuchal muscles.

The volatile anesthetic desflurane was administrated at stepwise decreasing concentrations at 6, 4, 2, and experiments commenced 1 to 8 days after surgery. A 15-min equilibrium period was allowed to stabilize the anesthetic concentration between consecutive conditions. With each anesthetic concentration, neuronal activity was recorded first during a spontaneous activity for 20 min followed by a period with visual stimulation (light flashes delivered to the retina by transcranial illumination). The data obtained using visual stimulation were not used in this study. In one experiment that was performed at the beginning of the study, only 40 min of spontaneous activity was recorded (10 min per anesthetic concentration).

Our previous study with the same data discovered that, during desflurane anesthesia, most frequently at a 6% concentration, spontaneous spike activity was occasionally desynchronous while showing a low firing rate (Lee et al., 2020). This unexpected, paradoxical desynchronized brain state has not been reported before and contends with the generally presumed dose-dependent effect of anesthesia. Because this study is related to the typical signatures of anesthesia (slow oscillation and burst suppression), we excluded the desynchronization periods from the analysis. On average, 0.3, 10.3, 6.5, and 40% of the data were classified as a paradoxical desynchronized state in 0, 2, 4, and 6% desflurane, respectively.

#### Preprocessing

Extracellular potentials were recorded at a 30 kHz sampling rate (SmartBox; Neuronexus Technologies, Ann Arbor, MI, United States). For spike detection, the signals were median-referenced and high-pass filtered (300 Hz). Signals with an absolute value >10 SD were considered movement-related artifacts and automatically excluded from the analysis. The data were also visually inspected, and noticeable noise episodes were manually excluded. One experiment was excluded from the analysis because of severe noise contamination (thus, *n* = 7). A template-based spike sorting method, Spiking Circus (Yger et al., 2018), was used to identify single unit activity (SUA). Per animal, 36 ± 14 (mean ± SD) single units were obtained.

#### ON and OFF State Detection

In both the model and the experiment, neural firing often shows a transition between periods of sustained firing (ON period) and quiescence (OFF period). A visual inspection of the 2D network model suggested a strong association between the OFF period duration and the firing rate peak of an ensuing ON period. To identify the ON and OFF periods in anesthesia experiment data, a discrete-time hidden semi-Markov probabilistic model was used to infer the two states, the ON and OFF periods (Chen et al., 2009). A population spiking activity was detected by the summation of the spike activity of all recorded neurons and considered as a single stochastic point process. The rate of the point process was determined by the firing history of the population spiking activity and the discrete hidden state. The expectation maximization algorithm was used to estimate the parameters from the statistical model (Chen et al., 2009). The parameters for the model were chosen following a previous study (Jercog et al., 2017), namely, bin size: 10 ms, number of history bins: 2, history dependence weight: 0.01, transition matrix = p_OFF → ON_ = p_ON → OFF_ = 0.1, p_OFF → OFF_ = p_ON → ON_ = 0.9, rate during ON periods: 3, and rate difference during OFF and ON periods: −2. The algorithm gave the ON and OFF periods with a 10 ms time resolution. The algorithm was applied to data at a 6% desflurane concentration, during which slow oscillation and burst suppression patterns were pronounced.

In the 2D network model, the firing patterns during ON-OFF transitions are regular and similar across different cycles, and, thus, the periods are classified by simply applying a 1-Hz threshold to the averaged iFR (instantaneous firing rate) time series (50 ms rectangular window). Applying the same method as in the experimental data showed qualitatively similar results.

### Statistical Analysis

All the statistical analyses were conducted using a StatsModels library (www.statsmodels.org) in Python 3.8. For firing rate, synchronization, and the correlation between the OFF duration and the iFR peak of the following ON duration, the differences between different desflurane concentrations (anesthesia experiment) for different τ_*ATP*_ values were examined. We used a linear mixed model with a restricted maximum likelihood estimation. For experimental data, the desflurane concentrations (categorical independent variable) were used as a fixed effect, and the random effect included seven animals. For the simulation data, the different ATP production rates (categorical independent variable) were used as a fixed effect, and the random effect included 10 different simulations. A *P*-value of <0.05 was considered a significant difference.

## Results

### State Transition in the Mean-Field Model

We first analyzed the dynamics of a single neuron mean-field feedback model with varying ATP production time constants, τ_*ATP*_. The synaptic input for a single neuron is modulated proportionally to the own temporal firing rate of the neuron, which is updated in real-time with a 200-ms moving window.

**Figure 2:**
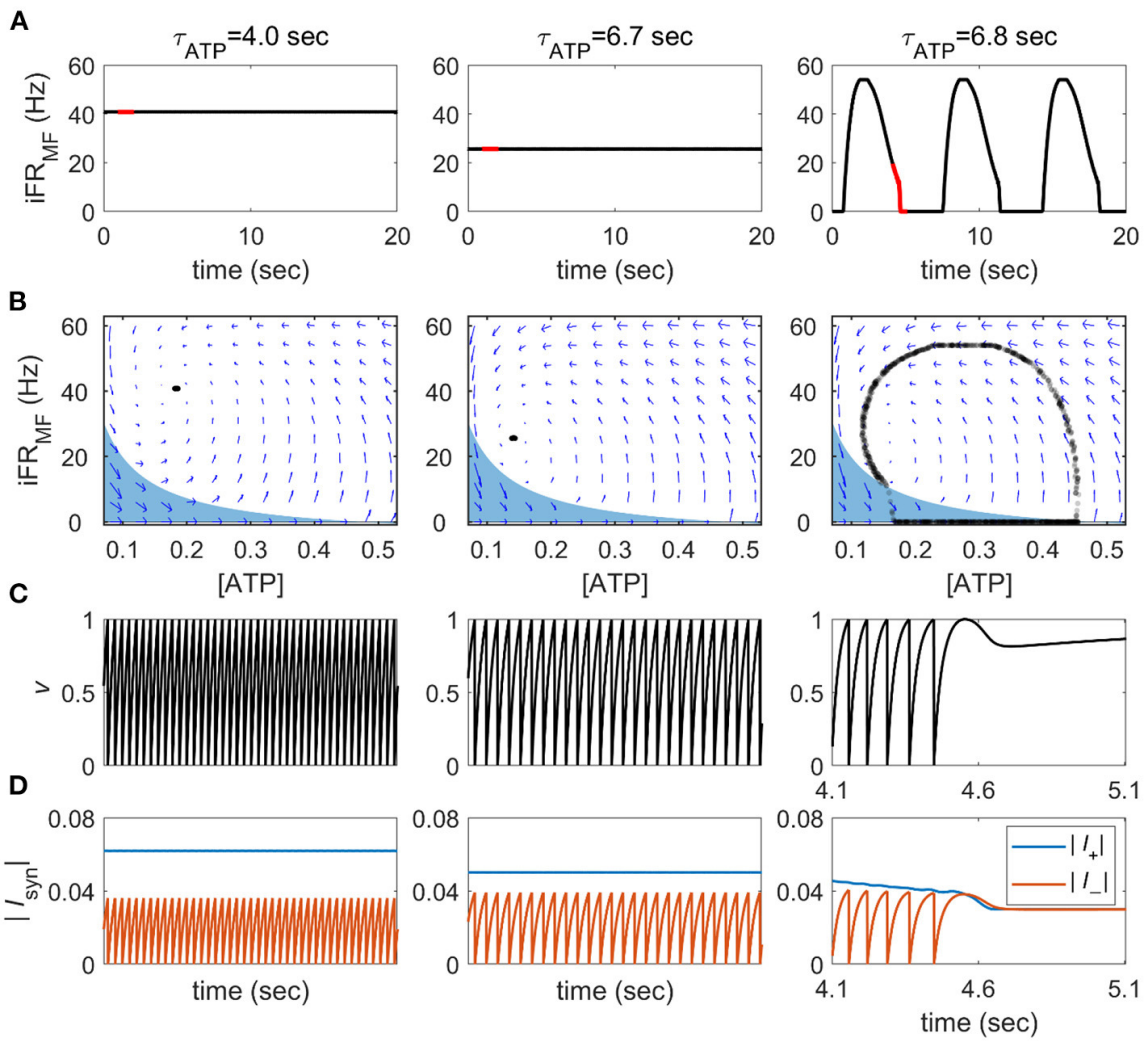
State transitions of the single neuron mean-field feedback model. **(A)** The instantaneous firing rate of the mean-field feedback model (iFR_MF_) was calculated from a 200-ms Hann moving window. The red line notes the time periods on panel **(C,D)**. **(B)** Phase plane representation for the averaged intracellular ATP concentration (ATP) and instantaneous firing rate. Blue arrows represent the numerically calculated vector field. The blue area shows a range where a spike cannot be generated, and 1,000 time points are randomly sampled from 40,000 time points during a 20 s **(C)** *v*-time course in the red line notes in panel **(A)**. **(D)** The amplitude of the sum of positive and negative currents in the red line notes in panel **(A)**.

Depending on the value of τ_*ATP*_, we could observe two distinct dynamical patterns. When the production rate of ATP rate was sufficiently high (τ_*ATP*_ = 4 s, Figure 2), the firing rate was high and almost constant (40.82 ± 0.02 Hz). When τ_*ATP*_ was increased (τ_*ATP*_ = 6.7 s, Figure 2), the firing rate decreased with slower *v* growth, and the fixed point moved to a lower firing rate (25.64 ± 0.04 Hz). However, there were no qualitative changes in *v* and [ATP] dynamics. As τ_*ATP*_ *was* increased further (τ_*ATP*_ = 6.8 s, Figure 2), the *I*_*ATP*_ term became more negative so that the fixed point lost its stability. That is, the positive currents (*I*_*app*_,*I*_*syn*_ terms) can be smaller than the negative ones (*I*_*ATP*_,*I*_*leak*_ terms), which leads to a failure to produce a spike (τ_*ATP*_ = 6.8 s, Figure 2D). Once a spike was not emitted, the positive feedback cycle (i.e., a past spike causes a future spike) was disrupted, and then an OFF period began.

The region satisfying this condition can be obtained analytically by substituting *C*_*feedback*_*I*_*syn*_ to *C*_*feedback*_ (= 0.4) × *single spike response* (= 2) × *iFR*_*MF*_ in current balance equations. At *v* = 1, where a spike occurs, the equation is represented as follows:

$$\begin{array}{l} 0.03-\frac{1}{38.75}-0.002\times \frac{1}{\left[\text{ATP}\right]}+0.4\times 2\times {\text{iFR}}_{\text{MF}}<0. \end{array}$$

When the system reaches this area, it fails to create a spike and converges quickly to an iFR = 0 line. During an OFF period, [ATP] recovers until a spike can be generated even with *I*_*syn*_ = 0; then, once the spike starts, the neuron bursts explosively because of the accumulated [ATP]. In this way, the pattern of spikes acquires alternating ON on and OFF phases, which is qualitatively similar to the slow oscillation and burst suppression under limited energy supply conditions. In summary, reduced neuronal excitation due to limited energy supply disrupts the positive feedback loop of spike activity, resulting in a failure of sustainable firing and a qualitative change in firing dynamics.

### 2D Network Model Exhibits Fragmented Slow Oscillation

**Figure 3:**
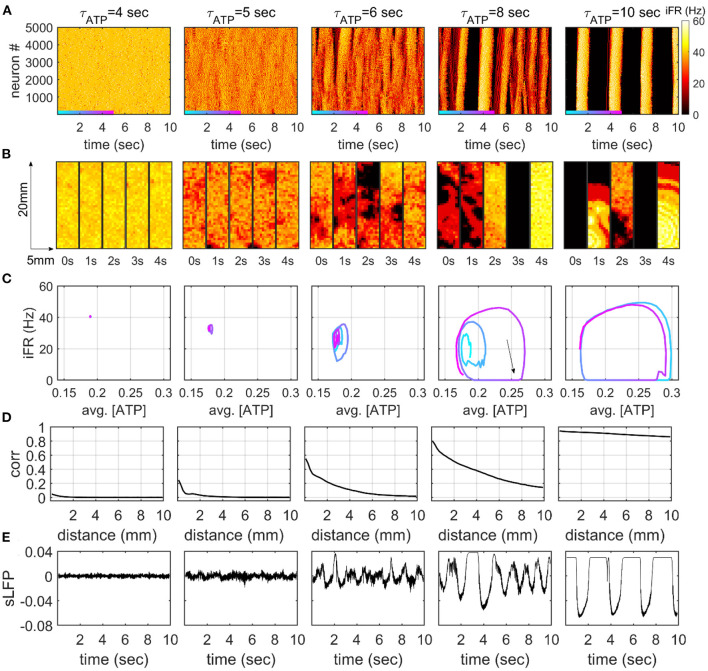
Dynamic firing patterns in the 2D network model. **(A)** The instantaneous firing rate (iFR) of 5,000 neurons. iFR is calculated with 50 ms rectangular moving windows. The order of neurons is determined by the y-axis locations of each neuron. **(B)** Snapshots of iFR in the 5 × 20-mm rectangular 2D space. The snapshots are obtained at five time points for each τ_*ATP*_ value. Each bin in the snapshot corresponds to a 500 × 500 μm square. The color represents iFR with the same color scale in panel **(A)**. **(C)** Phase diagrams for averaged [ATP] and iFR. [ATP] and iFR are averaged over 5,000 neurons with a 50-ms moving window. The colored trajectories represent the 5-s time windows in panel **(A)**. **(D)** Distance-dependent correlation of the iFR (50-ms window) of neurons. The Pearson correlation coefficients are averaged on the pairs with similar distances (200-μm bins). **(E)** The simulation of local field potential (sLFP) is calculated at the center of the 2D rectangular plane.

We simulated the dynamics of a neuronal network consisting of 5,000 excitatory neurons arranged in a 2D space with varying τ_*ATP*_. We observed characteristic dynamical patterns that corresponded to the two distinct patterns of the mean-field feedback model: a persistently high firing rate (τ_*ATP*_ = 4 s, Figure 3) and an ON-OFF cycle (τ_*ATP*_ = 10 s, Figure 3). In the constantly firing state, the neurons fired with near-zero correlations over all distances (τ_*ATP*_ = 4 s, Figure 3). For the ON-OFF cycle, a high correlation (>0.8) was maintained at a 10 mm distance, and all the neurons made burst together or stayed quiet together (τ_*ATP*_= 10 s, Figure 3).

Interestingly, our 2D model showed a novel dynamical pattern at an intermediate level of τ_*ATP*_. When ATP was lowered, the firing rate decreased, and the effect of excitatory feedback that comes from nearby excitatory neurons became dependent on spike timing. The firing rate started to fluctuate over time in the form of slow oscillation, as a consequence of the interaction between the network effect and the metabolism effect (Cunningham et al., 2006). In this state, neuron firing alternated between active and inactive periods, showing continuous waves with spatial fragmentation (τ_*ATP*_ = 5 s, Figure 3). That is, the spikes showed a high correlation at short range (<1mm), and they decayed drastically as the distance increased (τ_*ATP*_ = 5 s, Figure 3D). This spatially fragmented slow oscillation (FSO) formed small cycles on a phase plane defined with averaged [ATP] and iFR (τ_*ATP*_ = 5 s, Figure 3C), and the size of the cycle grew as τ_*ATP*_ increased (τ_*ATP*_ = 6 s, Figure 3C). The distance-dependent correlation (Figure 3D) and slow oscillation amplitude of the sLFP (Figure 3E) also showed a gradual increase as a function of τ_*ATP*_.

With further decreases in the production rate ATP, the FSO cycle became larger in amplitude, and globally synchronous silences and bursts began to appear for the first time (τ_*ATP*_ = 8 s Figure 3). A clear ON-OFF cycle pattern was triggered when the enlarged FSO cycle occasionally fell into global silence (iFR = 0 line, Figure 3C). As the system fell into a global silence, the neuronal network hardly got sufficient input to initiate firing, and the silence (OFF period) lasted for more than a second (τ_*ATP*_ = 8 s, Figure 3). This was consistent with the prediction of the mean-field model (Figure 2). During the OFF period, intracellular ATP was accumulated (black arrow, τ_*ATP*_ = 8 s, Figure 3C), which, in turn, enabled the neuronal network to initiate a series of firings with fewer triggering inputs. If sufficient input occurred because of probabilistic input currents, excitatory feedback induced a burst with an explosive consumption of the accumulated ATP from all neurons in the space. At the end of the burst, the trajectory in the phase plane was attracted by an FSO cycle or made a large turn and went back into the global silence again. In this way, the two cycles, the FSO cycle, and the ON-OFF cycle co-existed at the same τ_*ATP*_ in an alternating manner. Atτ_*ATP*_ = 10 s, the FSO cycle finally disappeared, and only the ON-OFF cycle remained.

### The Effect of Network Randomization on the Formation of FSO

Fragmented slow oscillation was not observed in the mean-field feedback model, but it occurred in the 2D network model with locally connected neurons. However, neural networks in the brain also have long-range connections in addition to the massive number of local connections, thereby displaying small-world topology, which will inevitably affect spatial synchronization. To examine if FSO appears in the presence of long-range connectivity, we re-ran the simulation on partially randomized networks after rewiring a certain portion (β) of connections (graph links).

**Figure 4:**
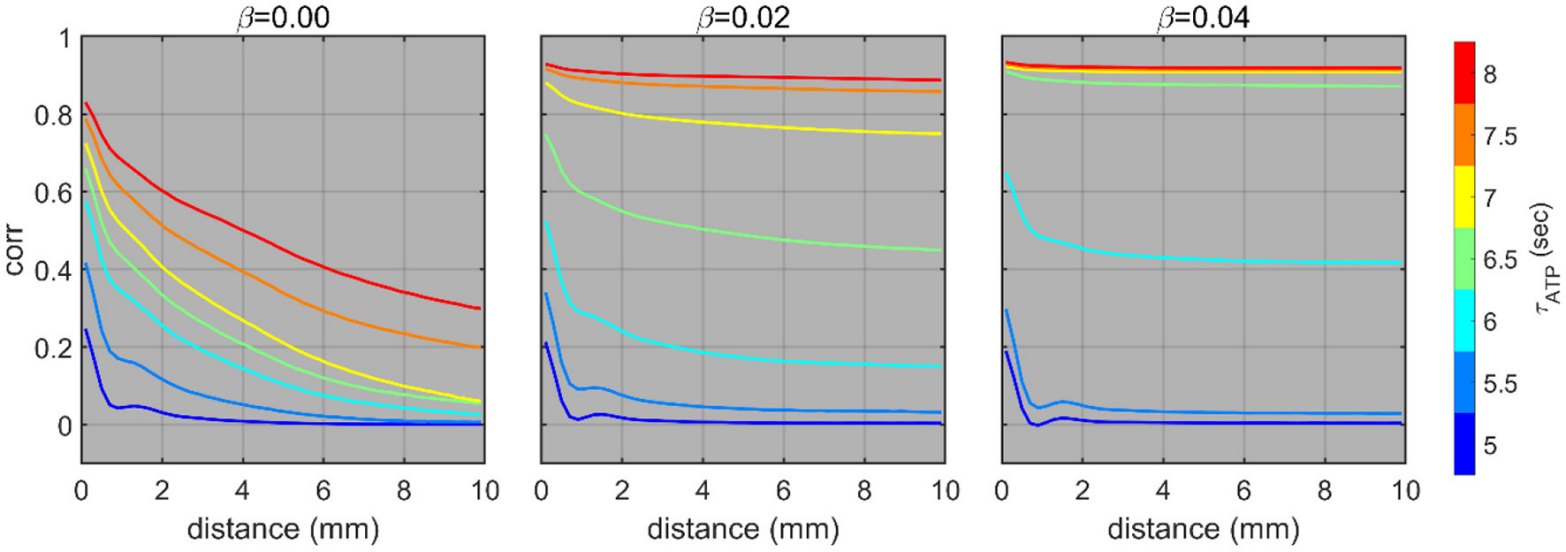
Distance-dependent correlations under different network rewiring probabilities (**β**). The distance-dependent correlations of the instantaneous firing rate (50 ms window) with different τ_*ATP*_ and **β**. The simulation was conducted 10 times in total, and the colored lines show the averaged results.

Fragmented slow oscillation is characterized by a high correlation at close distances that rapidly diminishes at further distances. As expected from the previous section, FSO was seen in various ranges of τ_*ATP*_ with β = 0. That is, the synchronization decayed as a function of distance. On the other hand, with β = 0.04, there was a jump from asynchrony to global synchrony. A decaying pattern was barely shown. This finding suggests that a small amount of random wiring (~4%) facilitates an abrupt transition from continuous to global synchrony as τ_*ATP*_ increases. Nevertheless, the sharp decaying correlations, that is, the evidence of FSO, were still observed in a range of τ_*ATP*_ under small network randomization (β = 0.02, Figure 4).

### Network Model With ATP Dynamics Predicts Firing Properties in Anesthesia

**Figure 5:**
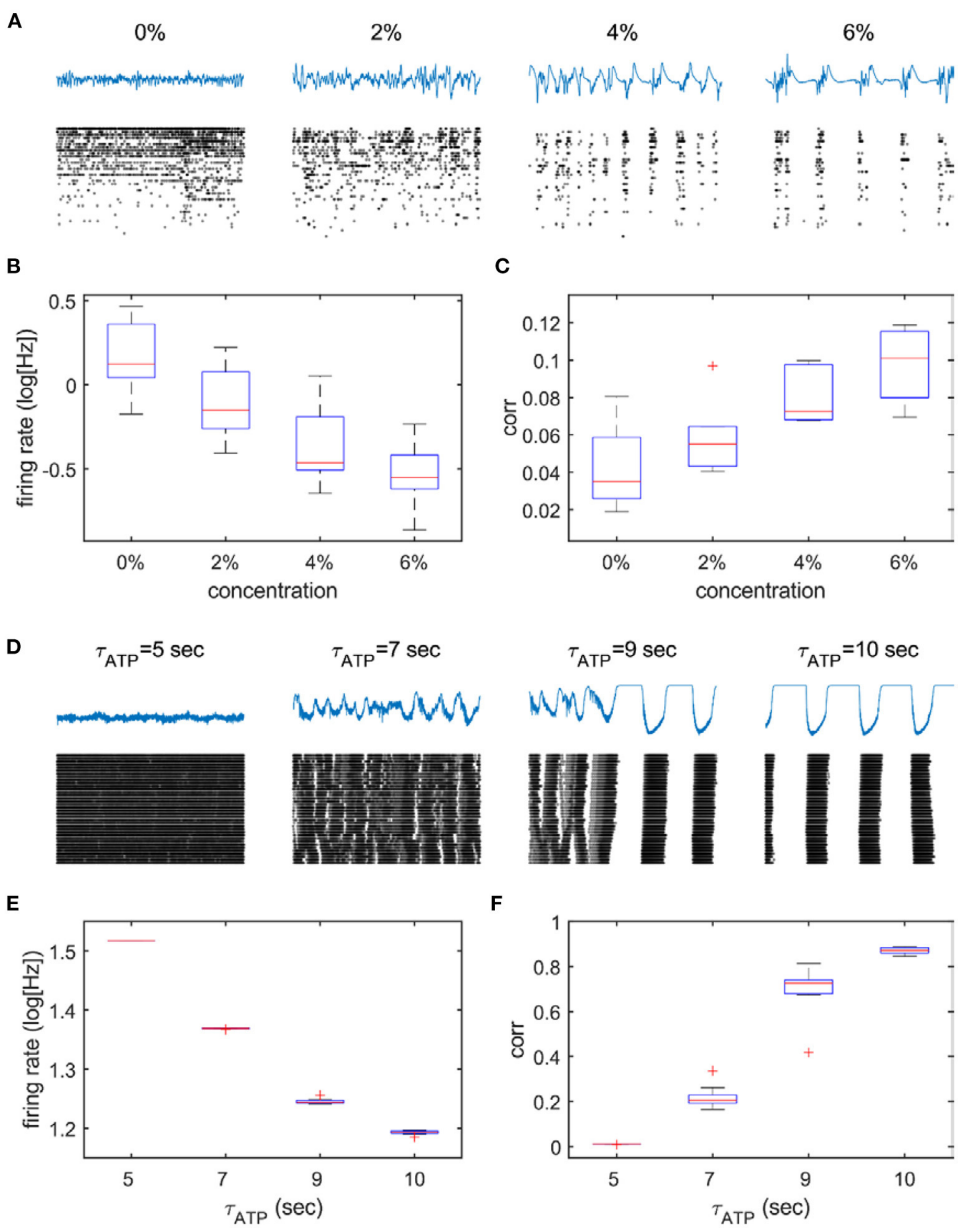
Suppressed spike activity and enhanced synchronization in the anesthesia experiment and the 2D network model. The upper panels **(A–C)** describe experimental data from different anesthetic concentrations, and the lower panels **(D–F)** describe model data from four selected parameters (τ_*ATP*_ = 5, 7, 9, and 10 s). **(A,D)** Raster plot (black dots) under different depths of anesthesia **(A)** and under increasing values of τ_*ATP*_ **(D)**. Note the increased bursting as anesthesia deepens and τ_*ATP*_ increases. The firing rate decreases **(B,E)** and **(C,F)** correlation increases monotonically.

Anesthesia is known to reduce ATP production and energy metabolism. We examined if model predictions with different values of τ_*ATP*_ *were in agreement with* the firing properties of cortical neurons under graded levels of anesthesia. As expected, the firing pattern of neurons changed in a similar way to that obtained from the 2D network model, that is, from high firing to sparse firing (Figures 5B,E) and from continuous firing to oscillation (Figures 5A,D). A statistically significant difference was found between 0% vs. all the other concentrations (*p* = 0.002, *p* < 0.001, and *p* < 0.001 for 0 vs. 2, 0 vs. 4, and 0 vs. 6% desflurane, respectively) and τ_*ATP*_ = 5 s vs. all the other τ_*ATP*_ values (*p* < 0.001 for all three comparisons). Simultaneously, the amplitude of LFPs increased, and the frequency slowed down. A pattern of burst suppression appeared in deep anesthesia (Figures 5A,D). Global synchronization, estimated by the averaged pair-wise correlation of spike trains of all recorded neurons, increased both in the anesthesia experiment and in the 2D network model (Figures 5C,F). A statistically significant difference was observed between 0 vs. 4–6% concentrations (*p* = 0.156, *p* = 0.003, and *p* < 0.001 for 0 vs. 2, 0 vs. 4, and 0 vs. 6% desflurane, respectively) and τ_*ATP*_ = 5 s vs. all the other τ_*ATP*_ values (*p* < 0.001 for all three comparisons).

**Figure 6:**
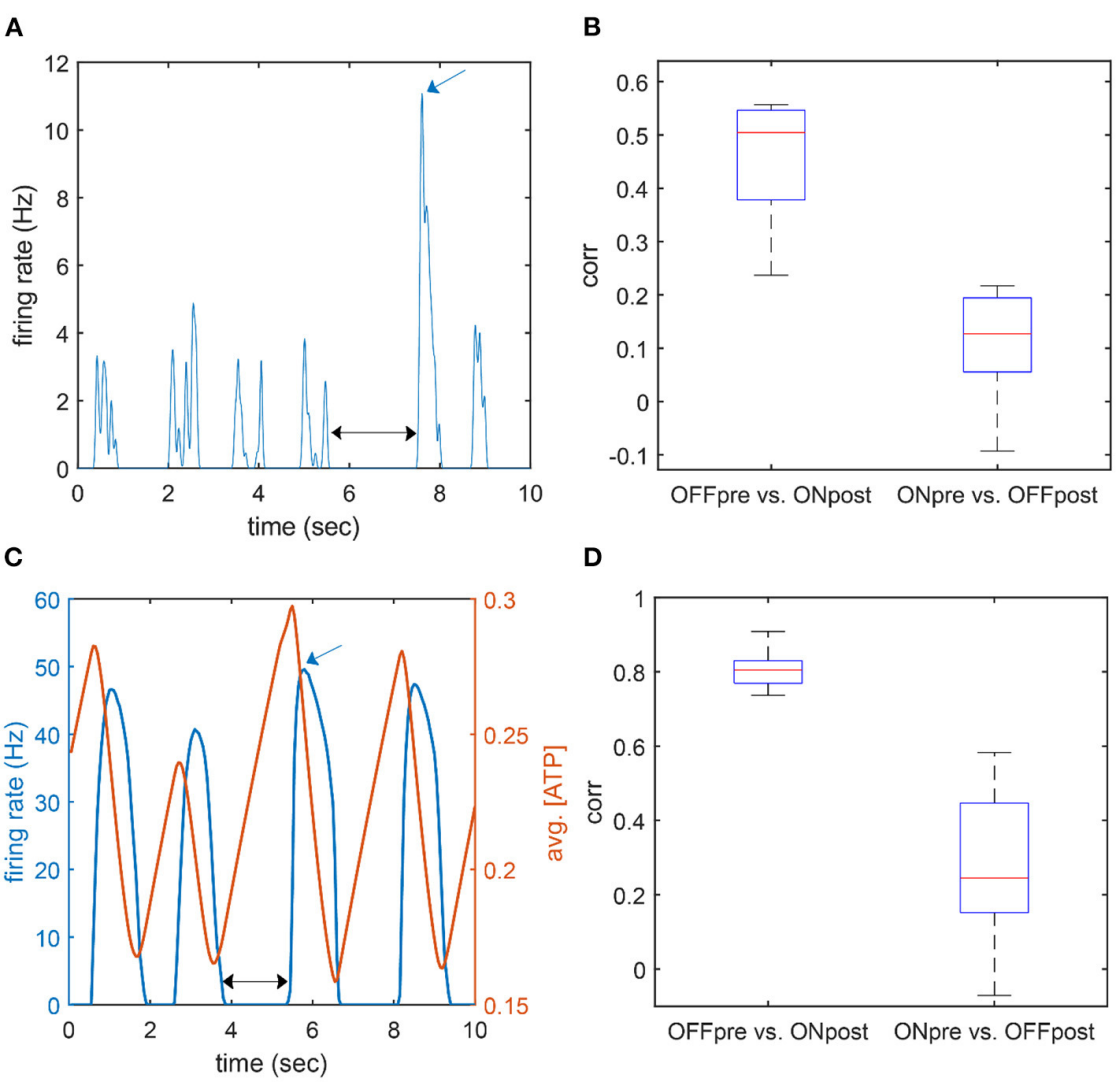
Correlation between peak firing rate and the adjacent OFF duration. **(A,C)** An example time course of the firing rate of population activity in the experiment (**A**; desflurane concentration: 6%) and the model (**B**; τ_*ATP*_ = 7 s). The black arrow marks an OFF duration (pre), and the blue arrow marks an on firing rate peak (post). **(B,D)** Correlation between OFF duration and ON firing rate peak. “OFF pre vs. ON post” indicates a correlation between the duration of the OFF period and the firing rate peak of the following ON period; “ON pre vs. OFF post” indicates a correlation between the firing rate peak of the ON period and the duration of the following OFF period. The former case showed a higher correlation.

As explained in the previous section, when τ_*ATP*_ is large, a longer duration of the off period potentiates ATP accumulation, which, in turn, elicits a larger burst of neuron firings in a short period of time (i.e., a large firing rate peak in the ON period). This suggests that there should be a positive correlation between the duration of the OFF period and the peak firing rate of the subsequent ON period. The correlation between the duration of the OFF period and the ensuing iFR peak showed a substantially high value both in the model (*r* = 0.808 ± 0.053) and empirical data (*r* = 0.456 ± 0.12) (Figure 6). On the other hand, the iFR peak of the ON period and the duration of the ensuing OFF period were essentially uncorrelated (*r* = 0.269 ± 0.189 in the model, r = 0.107 ± 0.108 in the empirical data). The correlation between the OFF duration and the ensuing iFR peak was significantly larger than the correlation between the iFR peak of the ON period and the ensuing OFF duration (*t*-test, experiment: *p* = 0.001; model: *p* < 0.001).

## Discussion

The goal of this study was to apply a neuronal network model to analyze the effect of reduced cerebral energy metabolism on the spike synchronization of neurons across a 2D space. We found that, when the production rate of ATP was sufficiently high, the neurons showed a fast and continuous firing pattern. When the energy metabolism was moderately reduced, neighboring neurons started to fire synchronously, reorganizing the firing pattern into FSO. When the ATP production rate was further decreased, neurons across the network eventually morphed into globally synchronous burst firing. The state transitions could then be explained by the failure of sustainable firing and the rebound firing after synchronized silence. These results were consistent with experimental data, which showed a low firing rate and increased synchronization under general anesthesia.

Metabolic rate has a crucial effect on the neuronal dynamics of the brain. The brain consumes most of its energy to support neuronal activities, such as synaptic transmission, the pumping of ions to maintain resting potential, and generating action potentials (Harris et al., 2012). Accordingly, the firing rate of neurons is highly correlated with the concurrent cerebral metabolic rate (Smith et al., 2002; Mäkiranta et al., 2005). The degree of neuronal spike activity can be regulated by the ATP-gated potassium channel that directly affects membrane potential as a function of intracellular ATP concentration (Yamada and Inagaki, 2002; Huang et al., 2007; Sun and Feng, 2013). The ATP-gated potassium channel has been included in neuronal models designed to explain the mechanisms of slow oscillation (Cunningham et al., 2006) and burst suppression (Ching et al., 2012) as a function of metabolic state.

In this study, we implemented a much more simplified neuron model, the leaky integrate-and-fire neuron but with the similar ATP dynamics as in other studies (Cunningham et al., 2006; Ching et al., 2012); this allowed us to simulate a relatively large number of neurons distributed in the 2D space. Thus, the effect of hypometabolism on spatial synchrony was examined. Our model study showed that a decrease in ATP production rate can enhance synchronization in the neuronal network, which starts with local weak synchronization, that is the FSO, that gradually evolves into strong global synchronization. Adding long-range connections to the network accelerated long-range distant synchronization. The higher the rewiring probability (β), the number of long-range links increased, and the number of short-range links decreased. This indicates that a neuron received more uncorrelated input from long-range links and that the excitatory feedback of the local network was reduced. Therefore, it would be difficult for the network to form local oscillations and the range of τ_*ATP*_, where FSO appears was reduced.

In addition, the mean-field feedback model, with which the structure of the network was approximated, showed an abrupt transition without an FSO-like pattern. The results suggest that the presence of local excitatory feedback, generated by nearby excitatory neurons in the neuronal network, plays a key role in the formation of FSO. Therefore, the change in the spatial synchronization of slowly oscillating neuronal dynamics under hypometabolic conditions can be a phased transition rather than an abrupt one due to the local network effect.

Slow oscillation (0.1–1 Hz) in the brain is characterized by rhythmic up and down phases and is dominantly observed in sleep and anesthesia (Steriade et al., 1993; Chauvette et al., 2011). The slow oscillation has been considered as a mostly cortical phenomenon as shown by its survival after thalamic lesions (Steriade et al., 1993) and many experiments that have demonstrated slow oscillations in cortical slices *in vitro* (Neske, 2016). Also, studies with a model of the cortical network (Compte et al., 2003; Cunningham et al., 2006) support the idea that the origin of the slow oscillation is the cortex. Consistent with previous findings, our model was able to predict the presence of slow oscillation in the absence of a subcortical modulation.

In our simulation with the mean-field model, the firing frequency of the neuron did not simply reduce as a function of τ_*ATP*_. It showed a qualitative dynamic change such that continuous firing changed to an oscillating firing pattern. The mean-field model explains that the transition is possible through the interaction between the positive feedback from the local excitatory network and the slow modulation by ATP consumption and production. That is, reduced positive feedback causes the failure of sustainable firing; during the silence period, ATP concentration slowly recovers, which, in turn, enables the emission of spikes again. This mechanism in itself is similar to the mechanism of the occurrence of oscillation by negative feedback, as in many studies, since a mechanism for spike frequency adaptation is suggested (Partridge and Stevens, 1976).

Burst suppression is characterized by alternating burst and suppression periods and is a prevalent phenomenon of deep anesthesia, hypoxic-ischemic comas, and hypothermia (Ching et al., 2012). Although many studies have been conducted to explain the characteristics of burst suppression (Swank and Watson, 1949; Steriade et al., 1994; Vijn and Sneyd, 1998; Ching et al., 2012; Lewis et al., 2013), the biophysical mechanism of the emergence of burst suppression remains unclear. In our model, the ON-OFF cycle, which corresponds to burst suppression, can emerge from diminished excitatory feedback because of the occurrence of a long-lasting silence. Based on our model predictions, we suggest that the growth of a slow oscillation cycle under weakened ATP production conditions enables an intermittent transition to burst suppression by increasing the possibility of a long-lasting silence. In addition, we can make a prediction for the intermediate state between slow oscillation and burst suppression. Sporadic large fluctuations, which reflect the intermittent occurrence of cycles (τ_*ATP*_ = 8 s, Figure 4), may be observed before burst suppression with increasing probability as the energy metabolism dwindles. The model predicted a strong association between the OFF duration and the iFR peak of the following on period; this was confirmed by experimental observation.

In this model, we assumed that cerebral hypometabolism affects neuronal activity, but in reality, they are linked in a closed loop. If neuronal activity is silenced directly, a commensurate decrease in cerebral metabolism follows. For example, anesthetics influence cerebral neuronal activity directly, through receptor-mediated and biophysical mechanisms (Hemmings et al., 2019), in addition to limiting intracellular high-energy phosphates because of the suppression of mitochondrial respiration. Studies in which positron emission tomography was performed revealed that whole brain metabolism is substantially diminished during the administration of propofol, sevoflurane, isoflurane, and halothane (Alkire et al., 1995, 1997, 1999; Kaisti et al., 2002). The metabolic suppression is correlated with simultaneous changes in quantitative EEG descriptors (Bispectral Index, total power, relative beta power, etc.) (Alkire, 1998). A causal link between metabolic and electrophysiological activities could be the abolished ATP production with anesthetics. Several commonly used anesthetics directly influence mitochondrial enzymes and metabolic pathways, reducing the production of ATP (La Monaca and Fodale, 2012). Abolished mitochondrial membrane potential under isoflurane, pentobarbital, or 1-phenoxy-2-propanol anesthesia can also inhibit mitochondrial ATP synthesis (Kishikawa et al., 2018). In line with these studies, our present model study shows that the ATP production rate could be a key regulator of the state transitions between continuous wake-like firing, globally asynchronous slow oscillation, and burst suppression. Thus, our findings, together with the above-cited studies, suggest that the suppression of neuronal activities due to diminished metabolism may be a principal mechanism for state transitions in general anesthesia.

We observed different degrees of spatial synchronization of slow activities in our model. Burst suppression has been known to be a predominantly synchronous phenomenon (Clark and Rosner, 1973; Lewis et al., 2013). On the other hand, recent experimental studies suggested that anesthetic-induced slow oscillations are asynchronous across the cortex (Lewis et al., 2012; Flores et al., 2017). In our model, spike bursts corresponding to slow oscillations and burst suppression exhibit qualitatively different synchronization patterns. Simulated slow oscillations appear globally desynchronized and form continuous waves with local up and down states. On the other hand, during burst suppression, the long-lasting silence acts as a bottleneck and, thereby, temporally aligns the rebound firing of neurons, enabling global synchronization. In this way, the suppression of spike activity caused by diminished ATP production can lead to enhanced synchronization without any modification of the physical connectivity between neurons.

## Limitations

First, the firing rate in our model network was uniformly distributed across neurons, distinct from many experimental studies, in which firing rate distribution follows a log-normal distribution (Buzsáki and Mizuseki, 2014). The uniformity in our model originated from the homogeneous degree distribution of the lattice-like model network. In this sense, our model might represent only a small portion of neurons with many and strong synaptic connections. However, because the synchronization of a highly inhomogeneous neuronal network is dominated by a small subset of high-degree nodes (Grinstein and Linsker, 2005), the overall dynamics would not be dramatically changed by additional neurons with less and weak synaptic connections were taken into account in the model system.

In addition, the leaky integrate-and-fire model, an extremely simplified neuron model, has limitations to the full reflection of the on-linear interaction of the actual neuron network. Consequently, we could not explain some spike characteristics, such as the inter-spike interval distribution. We used only one type of pyramidal neuron in our model, but there are numerous important factors in the real brain that we did not consider in this study. In particular, inhibitory neurons are known to play many important roles in synchronization (White et al., 1998; Steyn-Ross et al., 2013). A modeling study about the propagation of slow oscillatory spike bursts on a cortical network suggested that the speed of wave propagation is dramatically increased with the blockage of inhibition (Compte et al., 2003). Deep anesthesia accompanied by burst suppression is characterized by paradoxical hyperexcitability to sensory stimuli (Hartikainen et al., 1995; Detsch et al., 2002; Hudetz et al., 2007), presumably due to diminished inhibition (Kroeger and Amzica, 2007; Ferron et al., 2009). Based on these studies, it appears that the presence of local inhibitory neurons would not refute our results under general anesthesia. The structure of the connection is also greatly simplified using only some statistical values, which may result in different dynamics when the specific non-random connection structure is considered. Therefore, more sophisticated models will be required in the future to better reproduce the experimental results.

We did not reproduce the alpha and beta oscillations, which are associated with sedative and paradoxical excitation states under anesthesia (Brown et al., 2010; Purdon et al., 2015). The alpha and beta oscillations were previously reproduced with a thalamocortical circuit and synaptic modification mechanisms (e.g., gamma aminobutyric acid, GABA, agonist effect) (Ching et al., 2010; Hindriks and van Putten, 2012, 2013; Ching and Brown, 2014). Importantly, neural activity in this frequency range depends on the type of anesthetic; e.g., propofol and dexmedetomidine show different EEG patterns in the alpha-beta range but show a similar increase in the delta band (Purdon et al., 2015). Thus, neural activities in the alpha-beta frequency range may be related to specific agents and dose-dependent mechanisms and may not be explained solely by the suppression of metabolism.

## Conclusions

Our neuronal network model predicts that a decrease in cerebral ATP production leads to a monotonically decreasing firing rate with a transition from constant firing to locally synchronized firing followed by globally synchronized on-off alternate firing, consistent with experimental results. The model provides a framework for the further investigation of the biophysical mechanisms of the metabolism-dependent state transitions of neuronal networks.

## Data Availability Statement

The raw data supporting the conclusions of this article will be made available by the authors, without undue reservation.

## Ethics Statement

The animal study was reviewed and approved by Institutional Animal Care and Use.

## Author Contributions

PJ designed the study, performed the modeling, analyzed the data, and wrote the manuscript. HL analyzed the data, performed the statistical test, wrote the manuscript, and guided the whole research. SW performed the experiment and edited the manuscript. SK edited the manuscript and guided the whole research. AH interpreted the data, edited the manuscript, and supervised the whole research. All authors contributed to the article and approved the submitted version.

## Funding

The research reported in this publication was supported in part by the Center for Consciousness Science, Department of Anesthesiology, University of Michigan Medical School, Ann Arbor, Michigan, the United States, where the first author was a visiting scholar. This study was also supported in part by the National Research Foundation of Korea Grant funded by the Korean Government (NRF-2020R1F1A1076454).

## Conflict of Interest

The authors declare that the research was conducted in the absence of any commercial or financial relationships that could be construed as a potential conflict of interest.

## Publisher's Note

All claims expressed in this article are solely those of the authors and do not necessarily represent those of their affiliated organizations, or those of the publisher, the editors and the reviewers. Any product that may be evaluated in this article, or claim that may be made by its manufacturer, is not guaranteed or endorsed by the publisher.
